# B7-H3 chimeric antigen receptor-modified T cell shows potential for targeted treatment of acute myeloid leukaemia

**DOI:** 10.1186/s40001-023-01049-y

**Published:** 2023-03-20

**Authors:** Shuangshuang Fan, Tian Wang, Fengtao You, Tingting Zhang, Yafen Li, Cheng Ji, Zhichao Han, Binjie Sheng, Xiaochen Zhai, Gangli An, Huimin Meng, Lin Yang

**Affiliations:** 1 The Cyrus Tang Hematology Center, Soochow University, Suzhou, 215123 Jiangsu China; 2 Collaborative Innovation Center of Hematology, Soochow University, Suzhou, Jiangsu China; 3 State Key Laboratory of Radiation Medicine and Protection, School of Radiation Medicine and Protection, Soochow University, Suzhou, Jiangsu China; 4 PersonGen BioTherapeutics (Suzhou) Co., Ltd., Suzhou, Jiangsu China

**Keywords:** Chimeric antigen receptor-T cell, Immunotherapy, Acute myeloid leukaemia, B7-H3

## Abstract

**Background and aims:**

Chimeric antigen receptor (CAR)-T cell therapy is a novel type of immunotherapy. However, the use of CAR-T cells to treat acute myeloid leukaemia (AML) has limitations. B7-H3 is expressed in several malignancies, including some types of AML cells. However, its expression in normal tissues is low. Therefore, B7-H3 is ideal for targeted AML therapy.

**Materials and methods:**

First, we constructed B7-H3 CAR that can target B7-H3, and then constructed B7-H3-CAR-T cells in vitro, which were co-incubated with six AML cell lines expressing different levels of B7-H3, respectively. The toxicity and cytokines were detected by flow cytometry. In vivo, AML model was established in B-NSG mice to study the toxicity of B7-H3-CAR T on AML cells.

**Results:**

In vitro functional tests showed that B7-H3-CAR-T cells were cytotoxic to B7-H3-positive AML tumor cells and had good scavenging effect on B7-H3-expressing AML cell lines, and the cytokine results were consistent. In vivo, B7-H3-CAR-T cells significantly inhibited tumor cell growth in a mouse model of AML, prolonging mouse survival compared with controls.

**Conclusion:**

B7-H3-CAR-T cells may serve as a novel therapeutic method for the targeted treatment of AML.

**Supplementary Information:**

The online version contains supplementary material available at 10.1186/s40001-023-01049-y.

## Introduction

Acute myeloid leukaemia (AML) is a prevalent type of acute leukaemia. It is a highly heterogeneous malignant clonal disease that originates from the haematopoietic tissue. Patients have a high mortality rate, low long-term survival rate, and are prone to relapse, and thus, this disease greatly endangers human health and life [1, 2]. AML is a heterogeneous group of diseases and that there are several subgroups according to various classifications including immunophenotype, cytogenetics, the immunophenotype on the basis of which AML is divided because the classification is very complex and the treatment depends on it and various treatment protocols are applied [3, 4]. For decades, there has been little change in the treatment of AML, which still comprises chemotherapy and haematopoietic stem cell transplantation. Anthracyclines combined with cytarabine remain the classic chemotherapy regimen. Despite a relatively high remission rate, patients are at increased risk because of drug resistance and relapse [5–9].

Chimeric antigen receptor-T (CAR-T) cellular immunotherapy induces specific activation of T cells through antibodies that can recognize antigens on tumour cell surfaces, thereby avoiding the limitations of the major histocompatibility complex. Modified T cells can better recognize and destroy tumours than natural T cells. In recent years, CAR-T therapy has made great progress in cancer treatment and is considered one of the most promising tumour treatment methods. At present, CAR-T therapy has the greatest effect in acute lymphoblastic leukaemia (ALL) treatment [10–12], in which it is most widely used. CD19-targeting CAR-T treatment has achieved noteworthy results in the treatment of adult and child ALL [13, 14], with a remission rate of up to 90% [15]. CAR-T treatment of AML with antigens CD123 and CD33 also demonstrated good results. However, these two antigens are expressed in normal myeloid cells, which will inevitably result in toxic side effects in patients [16–22]. Therefore, identifying relatively safe and efficacious therapeutic targets is essential.

B7-H3, also known as CD276, was discovered in 2001. Studies have shown that B7-H3 can stimulate the expansion and destruction of T cells, and may selectively stimulate signal receptors on T cells [23]. B7-H3 is a tumour-associated antigen that plays an important role in tumour progression and metastasis. The survival rate of patients with negative B7-H3 protein expression is higher than that in patients with positive expression [24–27]. Several studies have shown that B7-H3 is abnormally expressed in human malignancies, such as melanoma, leukaemia, and breast and prostate cancer. The vast majority of cancer patients exhibited abnormally high expression (60–93%) of B7-H3 in cancer tissue, whereas B7-H3 expression in normal healthy tissue was low [28–32]. B7-H3 was highly expressed in AML, with the highest expression observed in the M3 and M5 subtypes [33].

Statistics show that approximately 40% of patients with AML express B7-H3 [34]. Moreover, some studies have shown B7-H3 expression in > 50% of cells in 31 pancreatic tumour specimens, but not in normal pancreatic tissue specimens [28]. B7-H3 expression is regulated by RNA transcription and is a surface immunoregulatory glycoprotein that inhibits natural killer cells and T cells. Although B7-H3 transcripts are widely expressed in human solid tumours and normal tissues, the B7-H3 protein is preferentially expressed in tumour tissue [29]. B7-H3 is expressed in only a few tissues and cells, including activated lymphocytes and tumour cells [35]. Therefore, although B7-H3 is expressed in part of AML, it is an excellent therapeutic target for the disease because it does not cause toxicity to the hematopoietic system.

It has been reported that B7-H3 is not expressed in immune cells [36]. In addition, most literature regarding B7-H3 has shown that B7-H3 is related to tumour progression and metastasis. More importantly, B7-H3 is highly expressed in tumour-related stromal cell fibroblasts and is associated with tumour neovascularization. Therefore, treatment targeting B7-H3 is expected to disrupt drug inhibition in the tumour microenvironment. This new target has attracted more attention in recent years [25]. Currently, several research groups have constructed B7-H3-CAR-T cells, which have shown encouraging tumour cell destruction in preclinical studies of various solid tumours such as pancreatic cancer, childhood neuroblastoma, and osteosarcoma [37–40]. In the current study, we constructed B7-H3-CAR-T cells with 4-1BB as a co-stimulatory domain carrying a safety switch, a truncated EGFR molecule.

## Materials and methods

### Cell lines and culture conditions

KG-1, MOLM-16, and Jurkat cells were purchased from the American Type Culture Collection (ATCC; Manassas, VA, USA). HEL, THP-1, HL-60, and AML5 cell lines were provided by Mr. Zhao Yun’s laboratory at the Tang Zhongying Hematology Research Center of Soochow University, Suzhou, China.

HEL, Jurkat, and AML5 cells were cultured in Roswell Park Memorial Institute (RPMI)-1640 medium (Hyclone,UT, USA) supplemented with 10% foetal bovine serum (FBS, Gibco, Grand Island, NY, USA). KG-1 cells were cultured in Iscove's modified Dulbecco’s media (Hyclone, UT, USA) supplemented with 10% FBS. Peripheral blood was drawn from healthy volunteers, and peripheral blood mononuclear cells were extracted by the Ficoll method. T cells were activated by Transact and cultured with TexMacsTMGMP medium (Hyclone, UT, USA) supplemented with interleukin (IL)-7 (155 U/mL; Novoprotein, China) and IL-15 (190 U/mL; Novoprotein, China). Extracted T cells were transduced after being activated for 48 h in vitro. Toxicity experiments were performed when cells proliferation numbers were sufficient. All cells were cultured in an incubator containing 5% CO_2_ at 37 °C.

### Construction of the B7-H3-specific CAR

The second-generation CAR was constructed as follows: the B7-H3-scFv sequence was cloned into a lentiviral vector, which contained a B7-H3-scFv, CD8 hinge and transmembrane region, a 41BB intracellular region sequence, a CD3ζ sequence, and a human EGFR sequence provided by PersonGen Bio Therapeutics (Suzhou, Jiangsu, China). Among them, EF1 represents the promoter, and 2A is a kind of self-cleavage sequence, which will be disconnected when translated into it. Proteins expressed by the same mRNA are separated before and after to produce two proteins, which are used to construct multi-cistronic carrier and realize co-translation of two proteins under the control of the same promoter. The role of 2A here is to achieve the co-expression of the front CAR protein and the truncated EGFR tagged protein behind. The promoter contains the signal peptide.

### Generation of B7-H3-specific CAR-modified T/Jurkat cells

A lentivirus was prepared according to conventional laboratory methods [41]. The CAR was constructed by gene synthesis and subcloned into a lentiviral plasmid and packaged into virus particles with infectious ability. T cells, 48 h post-activation, and Jurkat cells were placed in a 48-well plate (5 × 10^5^ cells/well). Thereafter, 50 µL of the virus was added, and TexMacsTMGMP/1640 medium was added to a final volume of 100 µL. Following transduction for 15 h, 1000 µL of TexMacsTMGMP/1640 medium was added to each well for culture. When the number of cells became sufficient, they were transferred to a 24-well plate or 12-well plate for culturing, and the CAR positive rate was tested when the number of cells was sufficient after approximately 10 days.

### Flow cytometry

Six AML cell lines (AML5, KG-1, HEL, HL-60, THP-1, and MOLM-16) were harvested and suspended in PBS (pH = 7.4). The cells were centrifuged at 4,000 rpm/min for 3 min, and the supernatant was discarded. An anti-B7-H3 antibody conjugated with allophycocyanin (APC) (1:1000) was added to the cells and incubated for 30 min. The cells were washed twice with PBS, and the expression of B7-H3 on the cell surface was detected by flow cytometry (Beckman Coulter, USA). Approximately 2 × 10^6^ cultured B7-H3-CAR Jurkat, B7-H3-CAR T, and T cells were harvested, incubated with 100 µL of 1:1000 diluted anti-EGFR antibody at 37 °C for 20 min, and washed twice with PBS. The positive rate of B7-H3-CAR Jurkat and B7-H3-CAR-T cells was analysed by flow cytometry [42].

Jurkat cells and B7-H3-CAR Jurkat cells were co-incubated with KG-1, HEL, and AML5 cells, and incubated with antibodies targeting CD25-APC and CD69-APC (BD Biosciences, USA) for 20 min, following which CD25 and CD69 expression levels were detected by flow cytometry.

### Western blotting

Approximately 1 × 10^6^ T cells and B7-H3-CAR-T cells were harvested. Lysates were separated using 8–12% sodium dodecyl sulphate–polyacrylamide gel electrophoresis and transferred onto polyvinylidene fluoride (PVDF) membranes (Millipore, MA, USA). Membranes were blocked with 5% skim milk for 1 h and incubated overnight with a mouse anti-human CD3ζ antibody (BD Biosciences, USA,) at 4 °C. Immune complexes were detected by incubating the PVDF membranes with a horseradish peroxidase-goat anti-mouse IgG antibody (Solarbio, Beijing, China) for 1 h at 37 °C. The protein bands were visualized with enhanced chemiluminescence reagents (Millipore, MA, USA).

### Cytotoxicity assays

Target cells (HEL, AML5, and KG-1) were resuspended in PBS and treated with 1 µL 5-(and-6)-carboxyfluorescein diacetate succinimidyl ester (CFSE) at 37 °C for 15 min. KG-1 cells served as a negative control group. Effector cells were added at a 1:1 effector:target (E:T) ratio, and cocultured with target cells for 48 h in a 24-well plate. Thereafter, cells were collected and resuspended in an equal volume of PBS. The cells were incubated with APC annexin V and 7-AAD (LK-AP105-100; Multisciences, Hangzhou, China), and the percentage of apoptotic cells was determined by flow cytometry (Beckman Coulter, USA) [43, 44].

### Cytokine secretion assays

The ability of T and B7-H3-CAR-T cells to produce IL-2, tumour necrosis factor (TNF), interferon γ (IFN-γ), and granzyme B in response to AML cells (HEL, AML5, and KG-1) was analysed using a cytometric bead array (CBA) system. The test fluid was the supernatant of previously killed tumour cells. The Human Granzyme B CBA Flex Set D7 Kit (BD Bioscience, USA, catalogue #560304), Human TNF Flex Set D9 Kit (BD Bioscience, USA), Human IL-2 Flex Set A4 Kit (BD Bioscience, USA), and Human IFN-γ CBA Flex Set E7 Kit (BD Bioscience, USA) were obtained from BD Biosciences [45].

### AML mouse xenograft model

Mice were housed under standard specific-pathogen-free conditions and all procedures met the requirements of the National Institutes of Health and Institutional Animal Care and Use Committee. Experiments were performed in accordance with protocols that were approved and authorized by the Animal Welfare and Ethics Committee of Soochow University. Ethical approval for this study was (Approval Number/ID: 201907A487, 201909A374 and 201912A149). The order of mouse grouping and experiments was randomly assigned. At first, all the mice in the experimental group were labelled with ear numbers and injected with tumour cells for unified observation. After that, the mice were randomly selected and injected with different effector cells. 6- to 8-week-old, female NOD-Prkdcs-cidIl2rgtm1/Bcgen (B-NSG) mice (Biocytogen, China) were engrafted with HEL cells via tail vein injection. Each mouse was injected with 5 × 10^5^ HEL-Luciferase-GFP cells, and the tumour burden was measured using an IVIS imaging system (IVIS-spectrum) after 2 or 5 days. Biofluorescence was recorded for 10 min after intraperitoneal injection of 150 mg/kg d-luciferin substrate. Mice were injected with an equal volume of effector cells in the tail vein [T cells, B7-H3-CAR-T cells, or phosphate buffered saline (PBS)]. Peripheral blood (100 μL), femoral bone marrow, and the organs which after erythrocyte lysis were incubated with anti-human CD45 antibody (BD Biosciences, USA). The cells were subsequently incubated for flow cytometry (Beckman Coulter, USA) analysis for the detection of tumour cells.

### Histology

After the mice were killed, the spleen and ovarian tissues were taken and embedded in paraffin for immunohistochemical analysis. After dewaxing and rehydrating, the paraffin sections were microwaved in a 0.01 M citrate buffer solution for 5 min and sealed with a methanol solution containing 0.3% hydrogen peroxide. The sections were incubated with an antibody targeting B7-H3 (1:300) followed by a biotin-labelled secondary antibody. Finally, DAB chromogenic solution was added to observe the infiltration of tumour cells in the tissues. Haematoxylin and eosin (HE) staining was performed after the paraffin sections were dewaxed and rehydrated to observe the pathological changes in tumour tissues.

### Statistical analysis

Statistical analyses were performed using GraphPad Prism5 Software (GraphPad Software Inc., La Jolla, CA, USA). Student’s *t*-test was used to compare values between two groups. *P* < 0.05 was considered to indicate a statistically significant difference. Kaplan–Meier curves were used to show survival of mice in each experimental group.

## Results

### Expression of B7-H3 in the AML cell lines

**Fig. 1:**
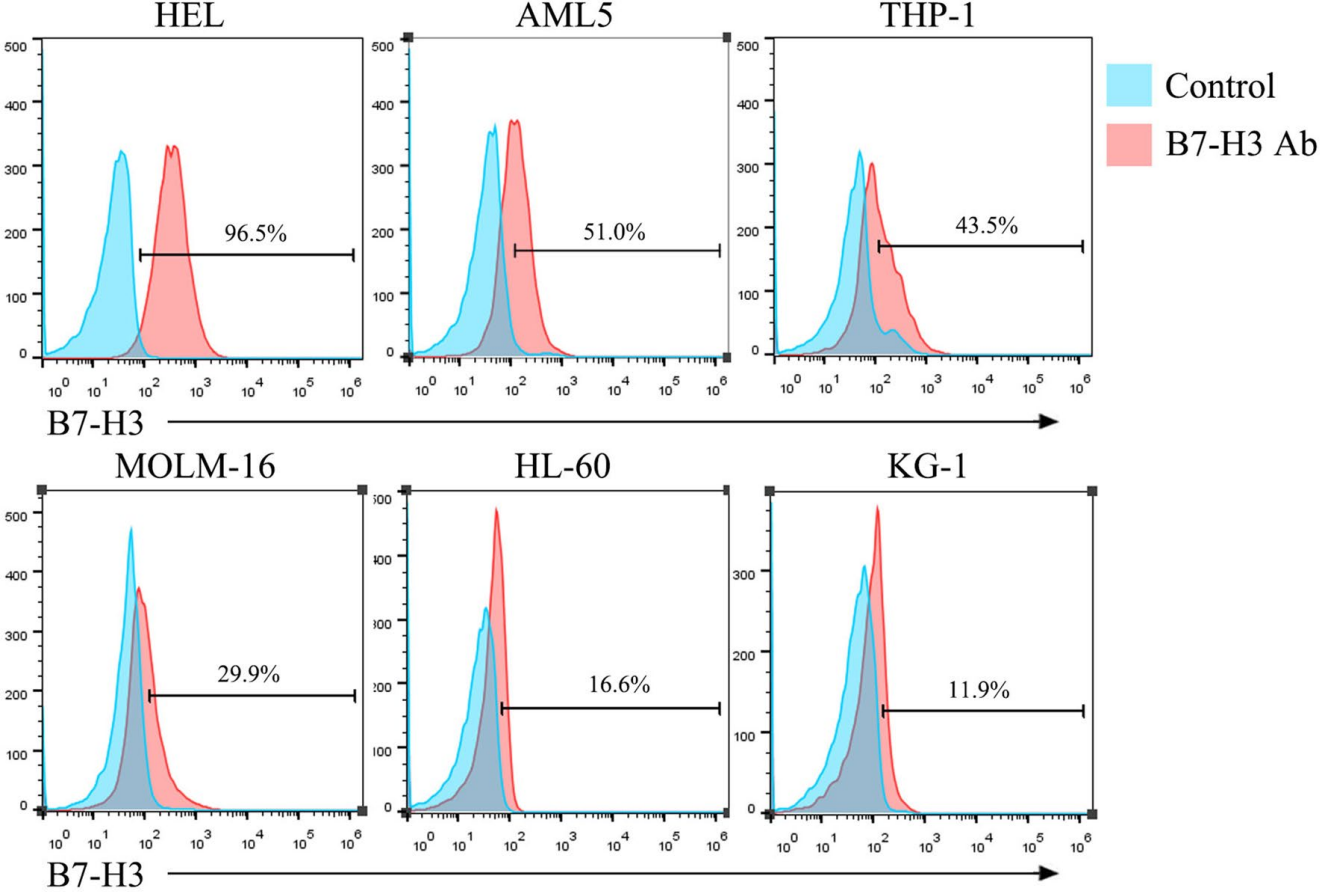
B7-H3 antigen expression rate on acute myeloid leukaemia (AML) tumour cell surfaces. Flow cytometry was used to detect B7-H3 expression on six AML tumour cells. In the figure, Control stands for control group

Six AML cell lines were divided into two groups, one of which was labelled with hAPC-B7-H3 antibody, while the other was not. B7-H3 expression was detected using flow cytometry. The expression rate of B7-H3 was 96.5, 51.0, 43.5, 29.9, 16.6, and 11.9% in HEL, AML5, THP-1, MOLM-16, HL-60, and KG-1 cells, respectively (Fig. 1). Finally, HEL, AML5, and KG-1 cells (high, medium, and low expression of B7-H3) were selected for subsequent experiments.

### Construction and specificity verification of B7-H3-CAR

**Fig. 2:**
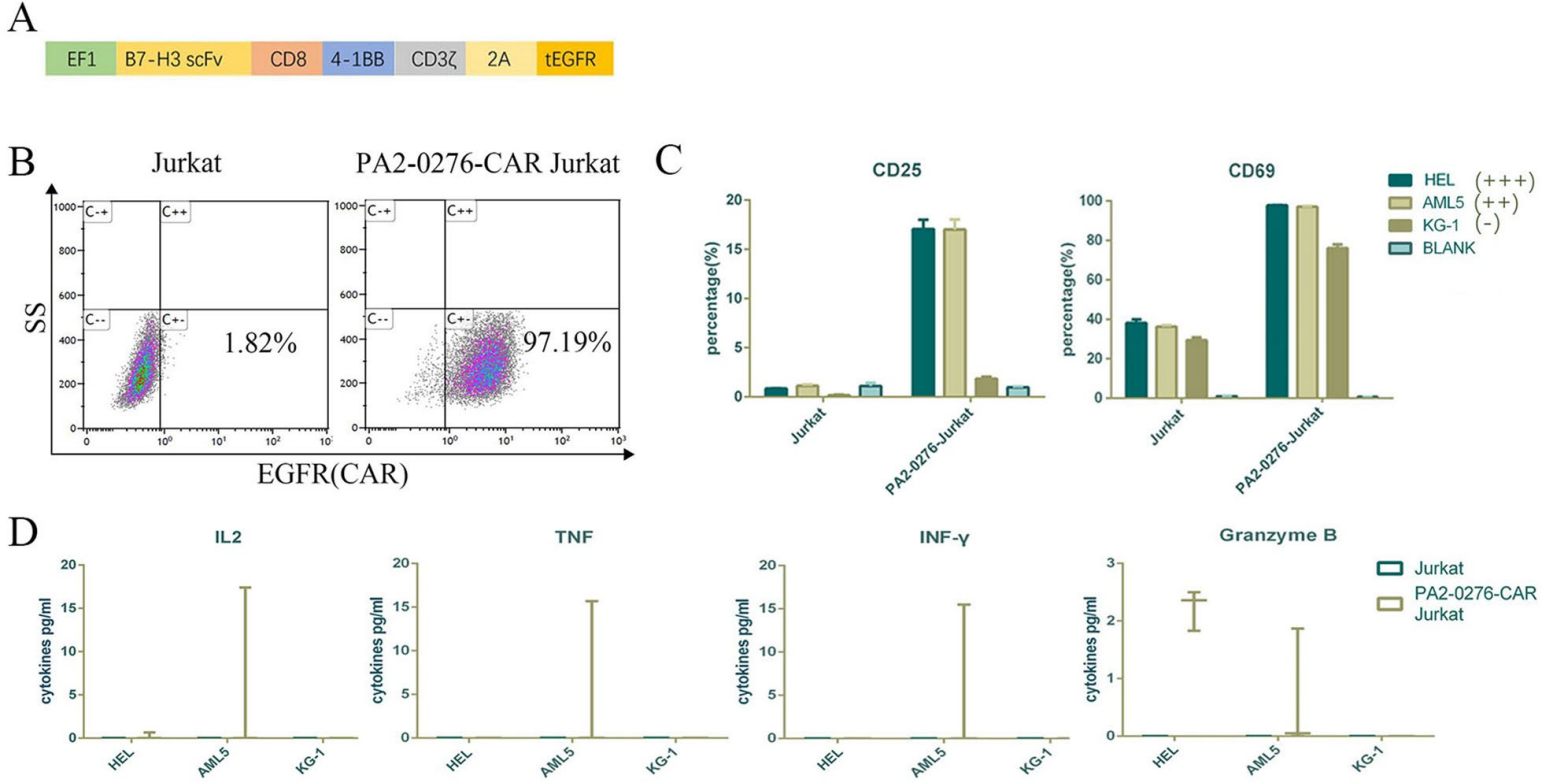
Structure and functional verification of B7-H3-CAR. **A** Schematic diagram of B7-H3-CAR, including the EF1 promoter, B7-H3 single-chain antibody, CD8 transmembrane structure, co-stimulation domain 41-BB, and intracellular signal domain CD3Z. **B** Flow cytometry analysis of B7-H3-CAR expression on the surface of Jurkat cells after transduction. **C** Expression of CD25 and CD69 on the surface of Jurkat and B7-H3-CAR-Jurkat cells was detected by flow cytometry using CD25-APC and CD69-APC antibodies after 48 h of 1:1 co-incubation with HEL, AML5 and KG-1 cells, respectively (*n* = 3 wells/group). In the figure, +++, ++ and + represent the high, medium and low expression of B7-H3 in cells, respectively. **D** The secretion of IL-2, TNF, INF-γ and granzyme B cytokines in the supernatant after the above co-incubation was measured

We constructed a CAR vector targeting B7-H3, which consisted of a second-generation CAR containing a B7-H3 single-chain antibody and a 4-1BB co-stimulatory domain, co-expressing EGFR, as a CAR detection tag (Fig. 2A). In addition, we used the Membrane Proteome Array method to test the specificity of the humanized B7-H3 antibody developed to assess for any off-target toxicity. Our antibodies were tested with a library containing more than 5,300 human membrane proteins, including 94% of all single-transmembrane proteins, multiple-transmembrane proteins, and GPI targeting proteins. Moreover, our humanized B7-H3 antibody specifically binds to only the B7-H3 protein, which indicates that the B7-H3 humanized antibody we developed will only specifically recognize the B7-H3 protein and not any other protein, thus producing no off-target toxicity, which further proves the specificity and safety of our humanized B7-H3 antibody. Following lentiviral transduction, B7-H3-CAR-Jurkat cells stably expressing B7-H3-CAR were constructed. The CAR positive rate of B7-H3-CAR-Jurkat cells was 97.19%, as revealed by flow cytometry (Fig. 2B).

The expression of CD25 and CD69 on the surface of B7-H3-CAR-Jurkat cells was higher than that on the surface of Jurkat cells after co-incubation with tumour cells with high expression of B7-H3 antigen. The cytokines related to T cell activation and tumor apoptosis were significant differences, and the AML5 cell line showed the most significant results in this result. There was no significant difference in the expression of CD25 and CD69 on the surface of B7-H3-CAR-Jurkat and Jurkat cells after co-incubation with tumour cells with a low expression of B7-H3 (Fig. 2C, D) CD69 is a marker for an early activation of T lymphocytes while CD25 is for late activation of T lymphocytes; both CD25 and CD69 are involved in the T lymphocytes proliferation, and their expression is correlated with the degree of immune response [46, 47]. The aforementioned results indicated that the constructed B7-H3-CAR-Jurkat cells were specifically activated by B7-H3-positive tumour cells.

### B7-H3-CAR-T eliminated B7-H3-positive AML cells in vitro

**Fig. 3:**
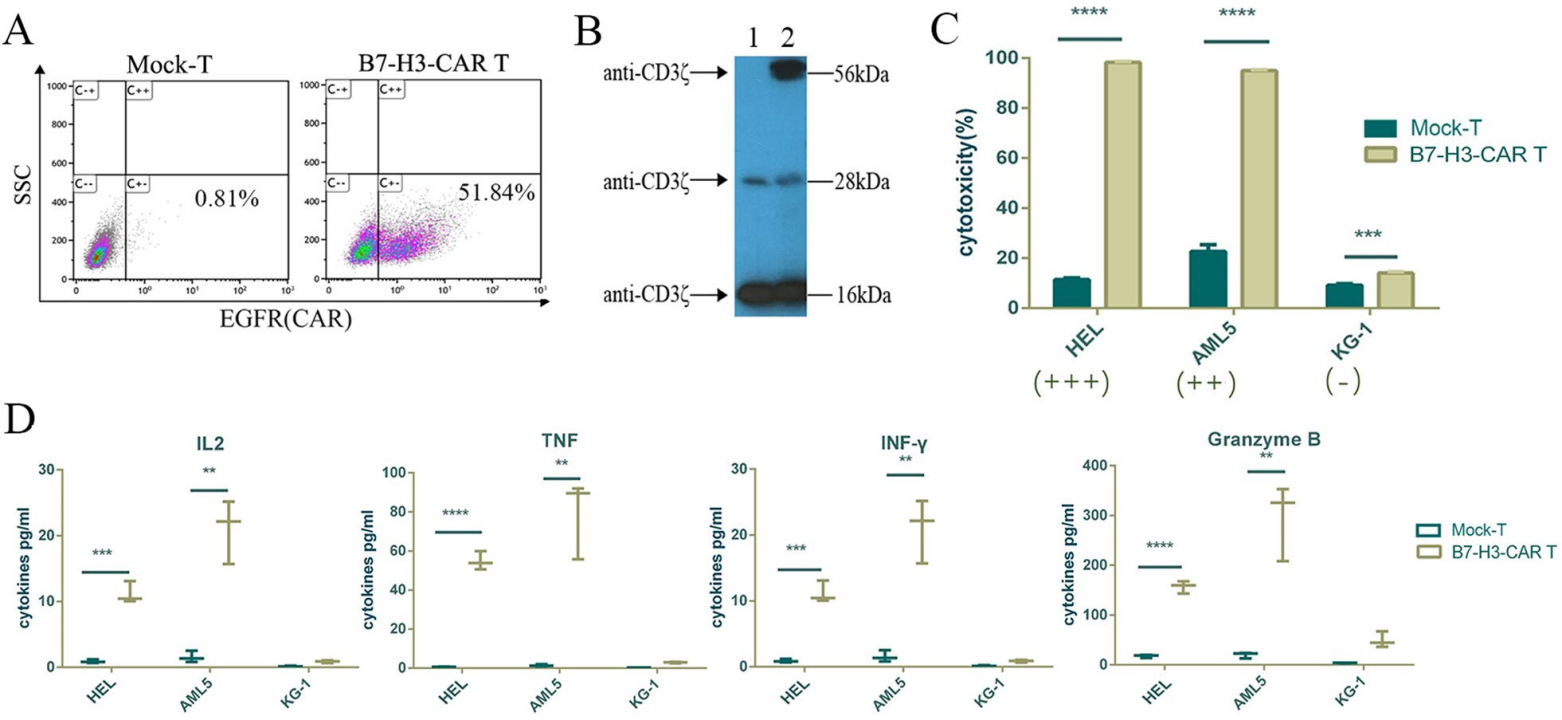
B7-H3-CAR-T cells have a specific elimination effect on B7-H3-positive acute myeloid leukaemia (AML) cell lines. **A** Flow cytometry analysis showed that the positive rate of B7-H3-CAR-T cells was approximately 51.84%. **B** Western bolt analysis of B7-H3-CAR expression in T cells. Proteins from T (lane 1) and B7-H3-CAR T (lane 2) cells were extracted and separated via sodium dodecyl sulphate–polyacrylamide gel electrophoresis. After incubation with CD3Z chain-specific monoclonal antibody and horse radish peroxidase-goat anti-mouse IgG, protein bands were detected by chemiluminescence. **C** After 48 h of incubation an E:T ratio of 1:1, the proportion of apoptotic target cells was detected by flow cytometry. ****P < 0.0001, *n* = 3. In the figure, +++, ++ and + represent the high, medium and low expression of B7-H3 in cells, respectively. D. Supernatant obtained following co-incubation, interleukin-2 (IL-2), Tumour Necrosis Factor (TNF), interferon gamma (INF-γ), and granzyme B cytokine secretion. ****P < 0.0001, ***P < 0.001, **P < 0.01 by Student’s *t* test. **C** and** D** represent one of three independent parallel experiments, each reaction was carried out in triplicate, *n* = 3 wells/group

B7-H3-CAR-T cells were prepared, and the positive rate was approximately 51.84%, as determined by flow cytometry (Fig. 3A). Western blotting was used to detect B7-H3-CAR fusion protein expression in B7-H3-CAR-T cells (Fig. 3B), which included a 16 and 28 kD endogenous CD3Z (lane 1 and lane 2) expressed by all T cells, and one 56 kD exogenous CD3Z (lane 2) expressed by CAR.

Flow cytometry was used to detect tumour cell apoptosis. The results showed that compared with T cells, B7-H3-CAR-T cells had a good cytotoxic effect on tumour cells with high and moderate expression of B7-H3. However, there was no significant difference in B7-H3 expression in tumour cells with low B7-H3 expression. This demonstrated that B7-H3-CAR-T cells exhibit a strong cytotoxic effect on AML cells expressing B7-H3 (*P* < 0.0001) (Fig. 3C). Thereafter, we tested cytokine secretions in the supernatant, and the results were consistent with the cytotoxicity results. The cytokine secretions of B7-H3-CAR-T cells co-incubated with tumour cells with high and moderate expression of B7-H3 were significantly higher than those of cells from other groups (*P* < 0.0001) (Fig. 3D). Further, it was suggested that the constructed B7-H3-CAR-T cells showed a high specificity in destroying AML tumour cells expressing B7-H3.

### B7-H3-CAR-T cells prolong the survival of the AML mouse xenograft model

To further evaluate the antitumor activity of B7-H3-CAR-T cells in vivo*,* we successfully constructed Luciferase-GFP-HEL cells (Additional file 1: Fig. S1A).

First, an AML mouse tumour model was established. Approximately 20 days after being injected with the Luciferase-GFP-HEL cells, the mice experienced depression, hind limb paralysis, and weight loss (Additional file 1: Fig. S1B). Bone marrow, peripheral blood, spleen, liver and kidney of mice were collected for flow cytometry analysis of tumor cell proportion (Additional file 1: Fig. S1C). The results revealed that mice injected with 5 × 10^5^ tumour cells via the tail vein could be used to successfully establish the AML model.

**Fig. 4:**
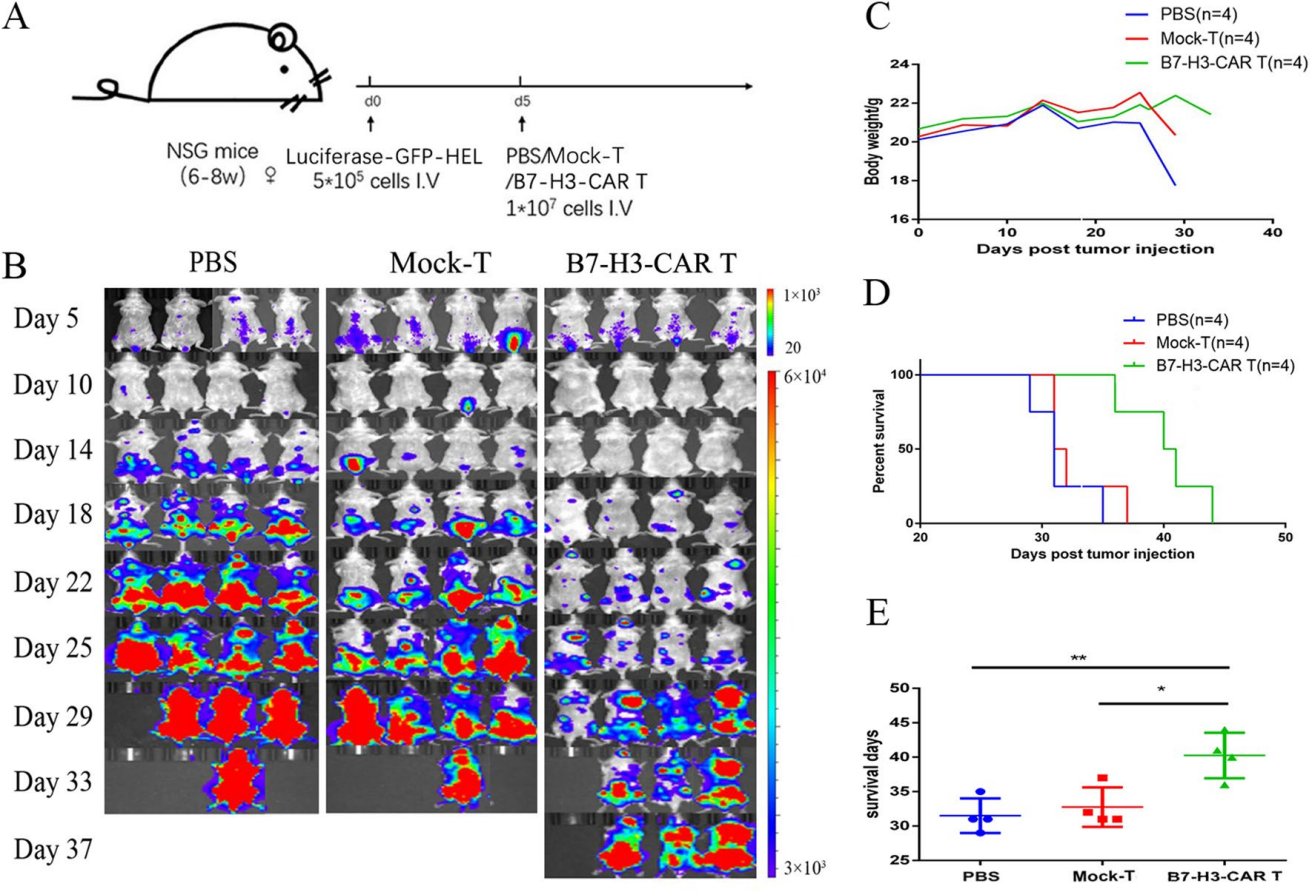
B7-H3-CAR-T cells exhibit antitumor activity in a xenograft mouse model of acute myeloid leukaemia (AML). **A** Schematic diagram of AML transplantation in B-NSG mice. Four female B-NSG mice (6–8 weeks old) in each group (PBS, T cell, and B7-H3-CAR-T cell groups) were injected with 5 × 10^5^ Luciferase-GFP-HEL cells via the tail vein, followed by 1 × 10^7^ effector cells in an equal volume on the day 5. An equal volume of PBS was injected into the mice in the PBS group. **B** In vivo imaging showing leukaemia progression in mice. **C** Average weight of the mice. **D** Mouse survival curve. **E** Mice survival statistics. **P < 0.01, *P < 0.05, *n* = 4 mice/group

Luciferase-GFP-HEL cells were injected into the tail vein of 12 B-NSG mice (Fig. 4A). The mice were divided into 3 groups (*n* = 4). Each mouse was injected with 5 × 10^5^ cells. In vivo imaging of mice detected a high proportion of tumour cells, which was equivalent to a model of advanced AML cases. Thereafter, the same amount of PBS/ T/B7-H3-CAR-T (positive rate is approximately 58.19%, as shown in Additional file 1: Fig. S2A) cells were injected into the tail vein. In vivo imaging showed that tumour progression in the mice in the B7-H3-CAR-T group was significantly weaker than in the other two groups (Fig. 4B).

The average weight of the mice in the PBS and T cell groups dropped sharply at approximately 30 days. However, the weight of the mice in the B7-H3 group was consistent (Fig. 4C); survival was monitored. The results showed that the survival time of the mice in the B7-H3-CAR-T group was significantly longer than that of those in the PBS and the T cell groups (*P* < 0.01) (Fig. 4D, E). The results suggested that B7-H3-CAR-T cells can effectively inhibit the growth of tumour cells in tumour-bearing mice. B7-H3-CAR-T cells have significant anti-tumour effects on B7-H3-positive AML cells in vivo.

### B7-H3-CAR-T cells can safely reduce tumour burden in xenograft mice

**Fig. 5:**
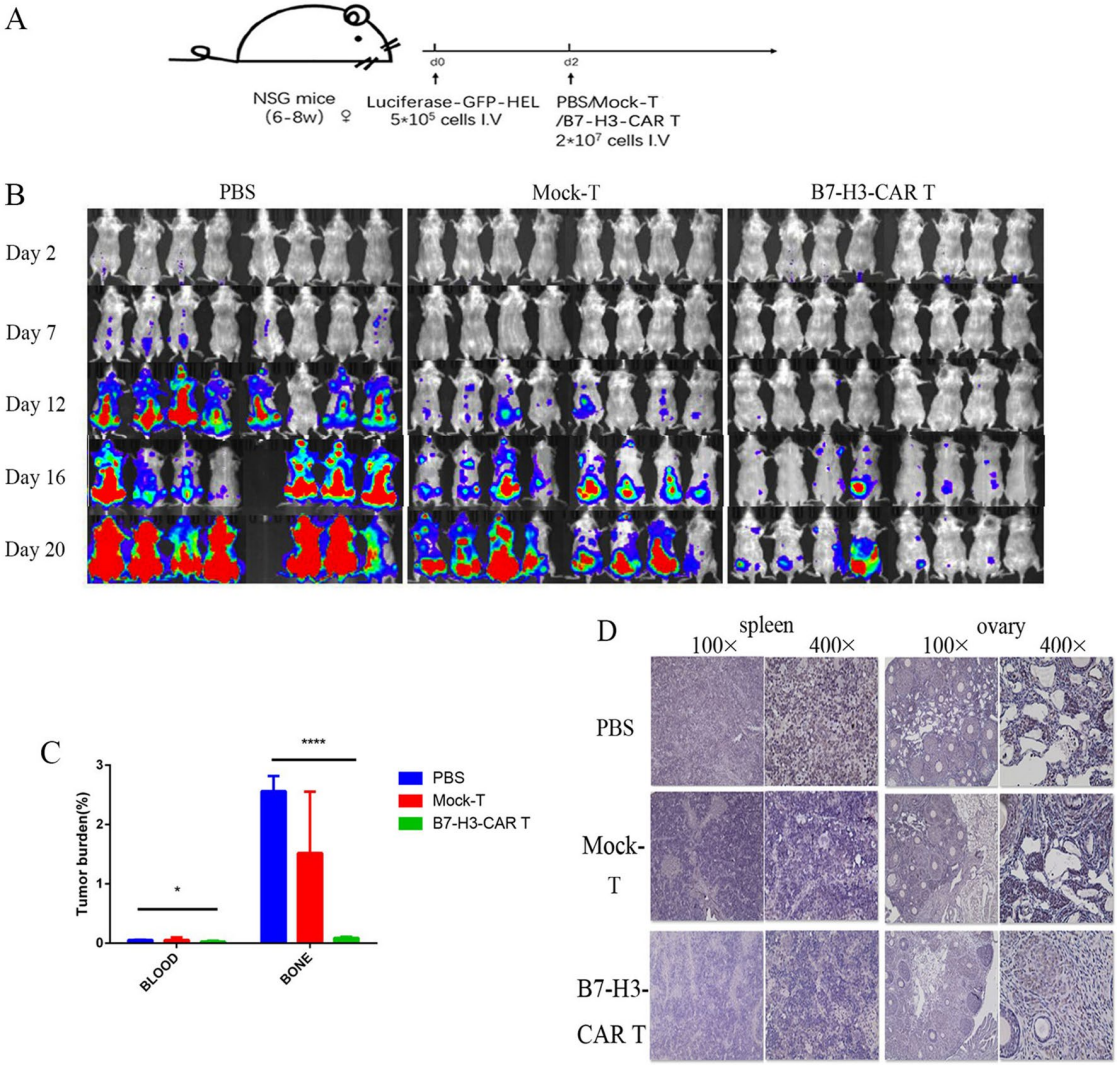
B7-H3-CAR-T cells can eliminate tumour cells from acute myeloid leukaemia (AML) xenograft mice. **A** Schematic diagram of the AML transplantation model in B-NSG mice. Three female NSG mice (6–8 weeks old) in each group (PBS, T cell, and B7-H3-CAR-T cell groups) were injected with 5 × 10^5^ Luciferase-GFP-HEL cells via the tail vein, and then injected with 2 × 10^7^ effector cells in an equal volume in the tail vein on the second day. An equal volume of PBS was injected into the mice in the PBS group. **B** In vivo imaging of mice showing disease progression. *n* = 8 mice/group. **C** After erythrocytes were lysed from mouse peripheral blood and bone marrow cells, cells were incubated with CD45 antibodies, and the proportion of tumour cells was detected by flow cytometry. ****P < 0.0001, *P < 0.05, *n* = 3 mice/group. **D** Immunohistochemistry of B7-H3 in the spleen and ovarian tissues of the mice showed that, compared with the B7-H3-CAR-T cell group, there was significant tumour cell infiltration in the PBS and T cell groups at a magnification of ×400 and ×100, *n* = 3 mice/group

To further demonstrate the antitumor effect of B7-H3-CAR-T cells in vivo, we constructed mouse models of AML by injecting tumour cells into the tail vein of mice (Fig. 5A). The day after tumour cell injection, in vivo imaging showed that tumour cells had infiltrated into the bone marrow, indicating that the model had been successfully established. Thereafter, we injected 2 × 10^7^ T cells and B7-H3-CAR-T cells (CAR positive rate was 52.82%, Additional file 2: Fig. S2B) into the mice, and performed live imaging every 4–5 days. The results showed that compared with the mice in the PBS and T cell groups, tumour growth in the mice in the B7-H3-CAR-T group was significantly suppressed (Fig. 5B).

At day 20, mice in the PBS group which untreated showed the same symptoms as in the first experiment, such as weight loss. So we euthanized all the mice and collected peripheral blood and bone marrow cells. The proportion of tumour cells was subsequently detected by flow cytometry (*P* < 0.0001) (Fig. 5C), and the results showed that the tumour burden of the mice in the B7-H3-CAR-T group was significantly lower than in the other two groups.

Immunohistochemical analysis of the spleen and ovarian tissues of the mice indicated significant tumour cell infiltration in the tissues of the mice in the PBS and T cell groups, whereas there was little or no tumour cell infiltration in the tissues of the mice in the B7-H3 group (Fig. 5D). Therefore, we demonstrated that B7-H3-CAR-T cells can effectively reduce the development of tumour cells in mice.

### B7-H3-CAR-T cells are not toxic to normal cells

The independent intellectual property scFv sequence used in this experiment is highly optimized for affinity and safety. Furthermore, it has a fast dissociation constant and a high killing activity. In addition, this sequence can recognize mouse B7-H3 molecules. Raji cells were overexpressed with mouse B7-H3 protein, then, T cells and B7-H3-CAR-T cells were co-incubated, at a ratio of 2:1, to kill Raji cells and mB7-H3-Raji cells. The results show that, compared with the control group, B7-H3-CAR-T cells can specifically kill only mB7-H3-Raji cells (Additional file 3: Fig. S3). Therefore, the safety of this sequence can be proved by detecting the activity of normal cells in mice.

**Fig. 6:**
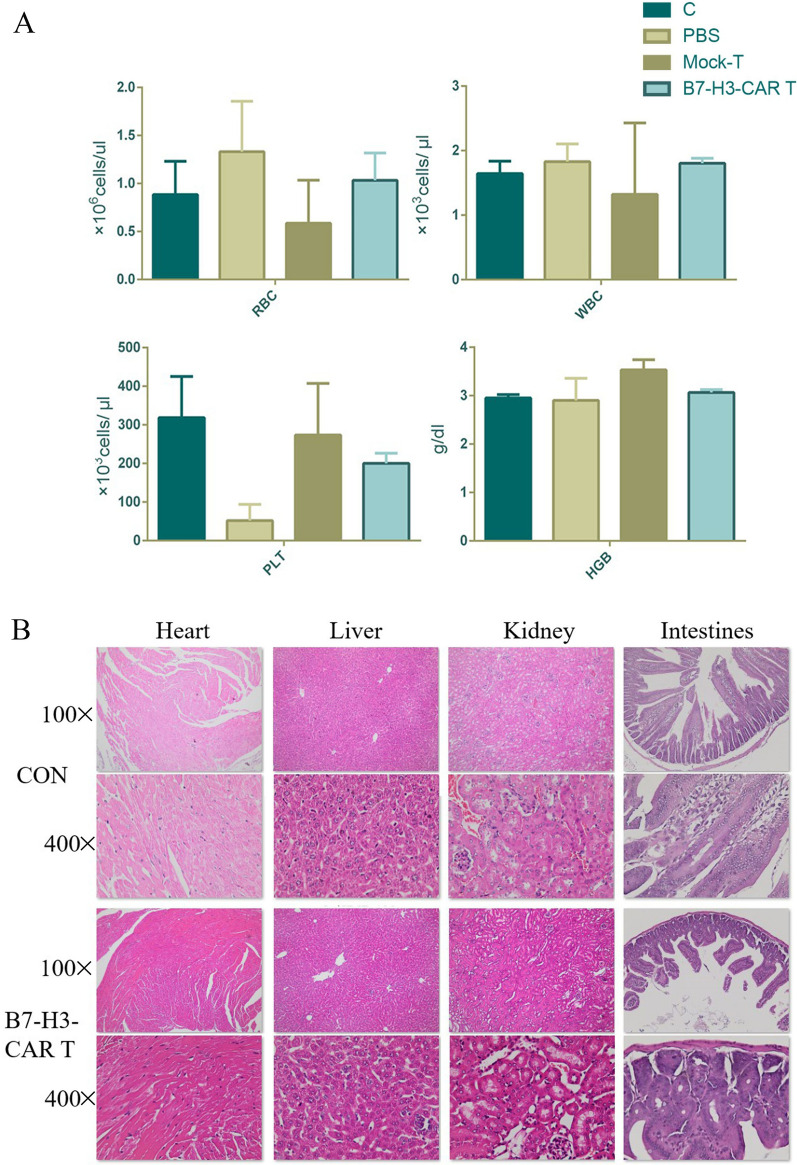
Safety verification of the B7-H3-CAR-T cells. **A** Statistics of red blood cells (RBC), white blood cells (WBC), platelets (PLT), and haemoglobin (HGB) after five classification of peripheral blood in mice. **B** The heart, liver, kidneys, and intestinal tissues of the mice were collected. HE staining was used to determine whether there were lesions in the tissues of the mice at a magnification of ×400 and ×100, *n* = 3 mice/group

We examined the peripheral blood of mice in the blank, PBS, T cell, and B7-H3-CAR-T cell groups. The results showed that there were no differences in white blood cells, red blood cells, platelets, or haemoglobin in the B7-H3-CAR-T mice compared with those in the control group (Fig. 6A). As we can be seen from the HE staining film, there was no tissue damage in the experimental group compared with the control group, and the cell size and morphology were consistent between the two groups. Therefore, compared with healthy mice, the B7-H3-CAR-T cell group showed no lesions in the heart, liver, kidneys, intestine and other vital organs (Fig. 6B). Collectively, the results suggested that that the B7-H3-CAR-T cells developed in the present study are a highly safe product.

## Discussion

AML is a refractory invasive hematopoietic stem cell malignancy [48]. In the past decade, the life expectancy of patients with AML has improved due to the development of new treatment methods. However, AML remains one of the most deadly and difficult to treat malignant cancers due to its high drug resistance [49–51]. Moreover, patients with AML have limited treatment options and a poor prognosis. Chemotherapy is not curative, and patients are prone to relapse. Therefore, new treatment methods are urgently needed [52–54]. CAR-T therapy has made great progress in both haematologic and solid tumours, and is considered to be one of the most promising tumour treatment methods [55, 56].

At present, the CAR-T treatment of AML requires bone marrow clearance before treatment because most of the targets are highly expressed in normal myeloid cells, which greatly complicates the treatment procedure and increases risks unnecessarily. Therefore, identifying an antigen that is selectively expressed in malignant cells is very important. B7-H3 was highly expressed in AML. Our experiments show that relying solely on T cells is not sufficient to eliminate tumour cells; However, B7-H3-CAR-T cells can effectively destroy B7-H3-expressing AML cells. CD25 and CD69 are activation markers and have been used to show cell proliferation in various models and on cells after and before treatment [57]. B7-H3-CAR-T cells also have a tumour clearance effect in mice. In vivo imaging tests showed that tumour cells in the mice in the B7-H3-CAR-T group were significantly lower than those in the PBS and T cell groups. Moreover, in cases of AML with a particularly rapid progression, B7-H3-CAR-T cells can significantly prolong the survival of mice and reduce the proportion of tumour cells in peripheral blood, bone marrow, and tissues. Therefore, the experiment proved B7-H3-CAR-T cells can be used for the treatment of patients with refractory AML who express the B7-H3 antigen.

In addition, to verify the safety of the B7-H3-CAR-T cells, we investigated whether they could cause off-target toxicity to normal cells in mice. Routine peripheral blood tests showed that the B7-H3-CAR-T cells did not have a toxic effect on normal cells. Moreover, HE staining results showed that B7-H3-CAR-T cells did not damage important organs in mice. Therefore, the results suggested that the B7-H3-CAR-T cells developed in the present study have a good safety profile. Two recent studies on the potential of B7-H3-CAR in the treatment of AML have well demonstrated the toxicity of B7-H3-CAR in AML cell lines and primary AML cells, laying a good foundation for the treatment of AML. Compared with these two studies, we used proprietary single-chain antibody sequences that not only demonstrated that B7-H3 significantly prolonged the survival of AML in mice, but also added in vivo data from peripheral blood, bone marrow, and tissue to further demonstrate the safety of B7-H3-CAR [58, 59].

In conclusion, we demonstrated that B7-H3-CAR-T cells can specifically remove AML tumour cells expressing the B7-H3 antigen in vitro and in vivo. Furthermore, the safety of the cells was demonstrated, laying the foundation for the clinical application of B7-H3-CART cells in AML treatment. Compared with CD33-CAR-T and CD123-CAR-T cells, which are the most frequently used cells at present, patients need to be clear myeloid cells in advance. The use of B7-H3-CAR-T cells may not only prolong the survival time of patients with relapsed refractory AML, but also simplify the application of CAR-T therapy in clinical practice and reduce risk in patients.

Future studies on CAR T may try to treat AML by combining it with hematopoietic stem cell transplantation (HSCT). The only treatment with long-term survival advantages for patients with AML is through the HSCT method, which, to date, there remains a relatively high recurrence rate and poor prognosis with it. The study found that CAR T treatment is a highly effective immune cell therapy known at this stage, therefore, it is best to combine the two. Patients can be pre-injected with CAR T cells prior to transplantation of allogeneic hematopoietic stem cells to help eliminate tumour cells in the body, thereby effectively reducing the disease burden of patients prior to transplantation, thus improving transplantation outcomes and survival rate [60].

In recent years, the development of CAR T treatment technology has been relatively rapid. There have been many research institutions in a relatively advanced position in CAR T treatment-related research and continue to maintain the current development momentum. We believe that CAR T cell products will soon benefit most cancer patients in the future.

However, several limitations exist. Only three mice were randomly selected from each group for flow cytometry, immunohistochemistry and HE staining of tumor burden in vivo. But the in vivo imaging data were sufficient to demonstrate a significant effect in the CAR T group. The primary AML cells were not used to verify the effect of B7-H3-CAR T. Because primary cells are difficult to obtain and ethical issues have not been solved for the time being, toxicity tests on primary cells cannot be conducted at present. Later studies in our laboratory will make up for this point, focusing on the toxic effect of B7-H3-CAR T on primary tumor cells.

## Conclusions

The B7-H3-CAR-T cells constructed by us have a great clinical application prospect as a novel therapeutic method for the targeted treatment of AML:

- We successfully established a novel second-generation chimeric antigen receptor (CAR) that could specifically target human B7-H3 including 4-1BB co-stimulatory domains, named B7-H3-CAR.
- B7-H3-CAR-T cells have significant anti-tumour effects on B7-H3-positive AML cells and can effectively reduce the development of tumour cells in vitro and vivo.
- The B7-H3-CAR-T cells developed in the present study are a highly safe product.

## Supplementary Information

**Additional file 1.** Established acute myeloid leukaemia (AML) xenograft model. **A.** Luciferase-GFP-HEL cells were detected by flow cytometry. **B.** Schematic diagram of the mouse AML model. A total of 5 × 10^5^ Luciferase-GFP-HEL cells were injected into the tail vein of 6–8-week old female B-NSG mice. **C.** Flow cytometry of tumour cells in the bone marrow, peripheral blood, spleen, liver, and kidneys of tumour-bearing mice.

**Additional file 2.** **A.** Flow cytometric detection of the B7-H3-CAR-T cell positive rate in the first mouse experiment. **B.** Flow cytometric detection of the B7-H3-CAR-T cell positive rate in the second mouse experiment.

**Additional file 3.** Apoptosis ratio of target cell detection following a 24h incubation under the effect-to-target ratio of 2:1.

## Data Availability

All data from this study are publishable.

## References

[1] Ghirga F, Mori M, Infante P (2018). Current trends in Hedgehog signaling pathway inhibition by small molecules. Bioorg Med Chem Lett.

[2] Chen W, Zheng R, Baade PD (2016). Cancer statistics in China. CA.

[3] Čolović N, Denčić-Fekete M, Peruničić M (2020). Clinical characteristics and treatment outcome of hypocellular acute myeloid leukemia based on WHO classification. Indian J Hematol Blood Transfus.

[4] Jurisić V, Pavlović S, Colović N (2009). Single institute study of FLT3 mutation in acute myeloid leukemia with near tetraploidy in Serbia. J Genet.

[5] Cortes JE, Heidel FH, Hellmann A (2019). Randomized comparison of low dose cytarabine with or without glasdegib in patients with newly diagnosed acute myeloid leukemia or high-risk myelodysplastic syndrome. Leukemia.

[6] Wei G, Ni W, Chiao JW (2011). A meta-analysis of CAG (cytarabine, aclarubicin, G-CSF) regimen for the treatment of 1029 patients with acute myeloid leukemia and myelodysplastic syndrome. J Hematol Oncol.

[7] Hackl H, Astanina K, Wieser R (2017). Molecular and genetic alterations associated with therapy resistance and relapse of acute myeloid leukemia. J Hematol Oncol.

[8] Narayan R, Blonquist TM, Emadi A (2019). A phase 1 study of the antibody–drug conjugate brentuximab vedotin with re-induction chemotherapy in patients with CD30-expressing relapsed/refractory acute myeloid leukemia. Cancer.

[9] Yanada M, Naoe T (2012). Acute myeloid leukemia in older adults. Int J Hematol.

[10] Brentjens RJ, Davila ML, Riviere I (2013). CD19-targeted T cells rapidly induce molecular remissions in adults with chemotherapy-refractory acute lymphoblastic leukemia. Sci Transl Med.

[11] Davila ML, Riviere I, Wang X (2014). Efficacy and toxicity management of 19-28z CAR T cell therapy in B cell acute lymphoblastic leukemia. Sci Transl Med.

[12] Lee DW, Kochenderfer JN, Stetler-Stevenson M (2015). T cells expressing CD19 chimeric antigen receptors for acute lymphoblastic leukaemia in children and young adults: a phase 1 dose-escalation trial. Lancet.

[13] Sadelain M (2015). CAR therapy: the CD19 paradigm. J Clin Invest.

[14] Gardner RA, Finney O, Annesley C (2017). Intent-to-treat leukemia remission by CD19 CAR T cells of defined formulation and dose in children and young adults. Blood.

[15] Maude SL, Frey N, Shaw PA (2014). Chimeric antigen receptor T cells for sustained remissions in leukemia. N Engl J Med.

[16] Mardiros A, Dos Santos C, Mcdonald T (2013). T cells expressing CD123-specific chimeric antigen receptors exhibit specific cytolytic effector functions and antitumor effects against human acute myeloid leukemia. Blood.

[17] Gill S, Tasian SK, Ruella M (2014). Preclinical targeting of human acute myeloid leukemia and myeloablation using chimeric antigen receptor-modified T cells. Blood.

[18] Pizzitola I, Anjos-Afonso F, Rouault-Pierre K (2014). Chimeric antigen receptors against CD33/CD123 antigens efficiently target primary acute myeloid leukemia cells in vivo. Leukemia.

[19] Kim MY, Yu KR, Kenderian SS (2018). Genetic inactivation of CD33 in hematopoietic stem cells to enable CAR T cell immunotherapy for acute myeloid leukemia. Cell.

[20] Kenderian SS, Ruella M, Shestova O (2015). CD33-specific chimeric antigen receptor T cells exhibit potent preclinical activity against human acute myeloid leukemia. Leukemia.

[21] Wang QS, Wang Y, Lv HY (2015). Treatment of CD33-directed chimeric antigen receptor-modified T cells in one patient with relapsed and refractory acute myeloid leukemia. Mol Ther.

[22] Petrov JC, Wada M, Pinz KG (2018). Compound CAR T-cells as a double-pronged approach for treating acute myeloid leukemia. Leukemia.

[23] Zhou Z, Luther N, Ibrahim GM (2013). B7-H3, a potential therapeutic target, is expressed in diffuse intrinsic pontine glioma. J Neurooncol.

[24] Cong F, Yu H, Gao X (2017). Expression of CD24 and B7-H3 in breast cancer and the clinical significance. Oncol Lett.

[25] Chen YW, Tekle C, Fodstad O (2008). The immunoregulatory protein human B7H3 is a tumor-associated antigen that regulates tumor cell migration and invasion. Curr Cancer Drug Targets.

[26] Liu H, Tekle C, Chen YW (2011). B7-H3 silencing increases paclitaxel sensitivity by abrogating Jak2/Stat3 phosphorylation. Mol Cancer Ther.

[27] Wu J, Wang F, Liu X (2018). Correlation of IDH1 and B7H3 expression with prognosis of CRC patients. Eur J Surg Oncol.

[28] Zhao X, Zhang G-B, Gan W-J (2013). Silencing of B7-H3 increases gemcitabine sensitivity by promoting apoptosis in pancreatic carcinoma. Oncol Lett.

[29] Xu H, Cheung IY, Guo HF (2009). MicroRNA miR-29 modulates expression of immunoinhibitory molecule B7-H3: potential implications for immune based therapy of human solid tumors. Cancer Res.

[30] Kraan J, Van Den Broek P, Verhoef C (2014). Endothelial CD276 (B7-H3) expression is increased in human malignancies and distinguishes between normal and tumour-derived circulating endothelial cells. Br J Cancer.

[31] Picarda E, Ohaegbulam KC, Zang X (2016). Molecular pathways: targeting B7-H3 (CD276) for human cancer immunotherapy. Clin Cancer Res.

[32] Dong P, Xiong Y, Yue J (2018). B7H3 as a promoter of metastasis and promising therapeutic target. Front Oncol.

[33] Guery T, Roumier C, Berthon C (2015). B7-H3 protein expression in acute myeloid leukemia. Cancer Med.

[34] Hu Y, Lv X, Wu Y (2015). Expression of costimulatory molecule B7-H3 and its prognostic implications in human acute leukemia. Hematology.

[35] Sun J, Mao Y, Zhang Y-Q (2013). Clinical significance of the induction of macrophage differentiation by the costimulatory molecule B7-H3 in human non-small cell lung cancer. Oncol Lett.

[36] Burvenich IJG, Parakh S, Lee F-T (2018). Molecular imaging of T cell co-regulator factor B7-H3 with (89)Zr-DS-5573a. Theranostics.

[37] Zhang J, Wang J, Marzese DM (2019). B7H3 regulates differentiation and serves as a potential biomarker and theranostic target for human glioblastoma. Lab Invest.

[38] Ma L, Luo L, Qiao H (2007). Complete eradication of hepatocellular carcinomas by combined vasostatin gene therapy and B7H3-mediated immunotherapy. J Hepatol.

[39] Luo L, Qiao H, Meng F (2006). Arsenic trioxide synergizes with B7H3-mediated immunotherapy to eradicate hepatocellular carcinomas. Int J Cancer.

[40] Tang X, Zhao S, Zhang Y (2019). B7-H3 as a novel CAR-T therapeutic target for glioblastoma. Mol Ther Oncolytics.

[41] Chen Y, You F, Jiang L (2017). Gene-modified NK-92MI cells expressing a chimeric CD16-BB-zeta or CD64-BB-zeta receptor exhibit enhanced cancer-killing ability in combination with therapeutic antibody. Oncotarget.

[42] Jurisic V, Bogdanovic G, Kojic V (2006). Effect of TNF-alpha on Raji cells at different cellular levels estimated by various methods. Ann Hematol.

[43] Zheng H, Zou W, Shen J (2016). Opposite effects of coinjection and distant injection of mesenchymal stem cells on breast tumor cell growth. Stem Cells Transl Med.

[44] Lei Z, Duan H, Zhao T (2018). PARK2 inhibits osteosarcoma cell growth through the JAK2/STAT3/VEGF signaling pathway. Cell Death Dis.

[45] Jurisic V (2020). Multiomic analysis of cytokines in immuno-oncology. Expert Rev Proteomics.

[46] Caruso A, Licenziati S, Corulli M (1997). Flow cytometric analysis of activation markers on stimulated T cells and their correlation with cell proliferation. Cytometry.

[47] Li T (2009). Pseudolaric acid B suppresses T lymphocyte activation through inhibition of NF-κB signaling pathway and p38 phosphorylation. J Cell Biochem.

[48] Dijk MV, Murphy E, Morrell R (2011). The proteasome inhibitor bortezomib sensitizes AML with myelomonocytic differentiation to TRAIL mediated apoptosis. Cancers.

[49] Naganna N, Opoku-Temeng C, Choi EY (2019). Amino alkynylisoquinoline and alkynylnaphthyridine compounds potently inhibit acute myeloid leukemia proliferation in mice. EBioMedicine.

[50] Bose P, Vachhani P, Cortes JE (2017). Treatment of relapsed/refractory acute myeloid leukemia. Curr Treat Options Oncol.

[51] Guinn BA, Mohamedali A, Thomas NS (2007). Immunotherapy of myeloid leukaemia. Cancer Immunol Immunother.

[52] Ranganathan P, Yu X, Santhanam R (2015). Decitabine priming enhances the antileukemic effects of exportin 1 (XPO1) selective inhibitor selinexor in acute myeloid leukemia. Blood.

[53] Becton D, Dahl GV, Ravindranath Y (2006). Randomized use of cyclosporin A (CsA) to modulate P-glycoprotein in children with AML in remission: Pediatric Oncology Group Study 9421. Blood.

[54] Shen W, Patnaik MM, Ruiz A (2016). Immunovirotherapy with vesicular stomatitis virus and PD-L1 blockade enhances therapeutic outcome in murine acute myeloid leukemia. Blood.

[55] Nakazawa Y (2019). Chimeric antigen receptor T-cell therapy for hematological malignancies. Rinsho Ketsueki.

[56] June CH, O'connor RS, Kawalekar OU (2018). CAR T cell immunotherapy for human cancer. Science.

[57] Vuletić AM, Jovanić IP, Jurišić VB (2015). In-vitro activation of natural killer cells from regional lymph nodes of melanoma patients with interleukin-2 and interleukin-15. Melanoma Res.

[58] Zhang ZL, Jiang CY, Liu ZY (2020). B7-H3-targeted CAR-T cells exhibit potent antitumor effects on hematologic and solid tumors. Mol Ther Oncolytics.

[59] Lichtman E, Du HW, Shou PS (2021). Preclinical evaluation of B7-H3-specific chimeric antigen receptor T cells for the treatment of acute myeloid leukemia. Clin Cancer Res.

[60] Wang X, Popplewell LL, Wagner JR (2016). Phase 1 studies of central memory-derived CD19 CAR T-cell therapy following autologous HSCT in patients with B-cell NHL. Blood.

