# Supplementary figures and images for: B7-H3 chimeric antigen receptor-modified T cell shows potential for targeted treatment of acute myeloid leukaemia
Source: Eur J Med Res. 2023 Mar 20;28:129. doi: 10.1186/s40001-023-01049-y (PMC10026503; doi:10.1186/s40001-023-01049-y)

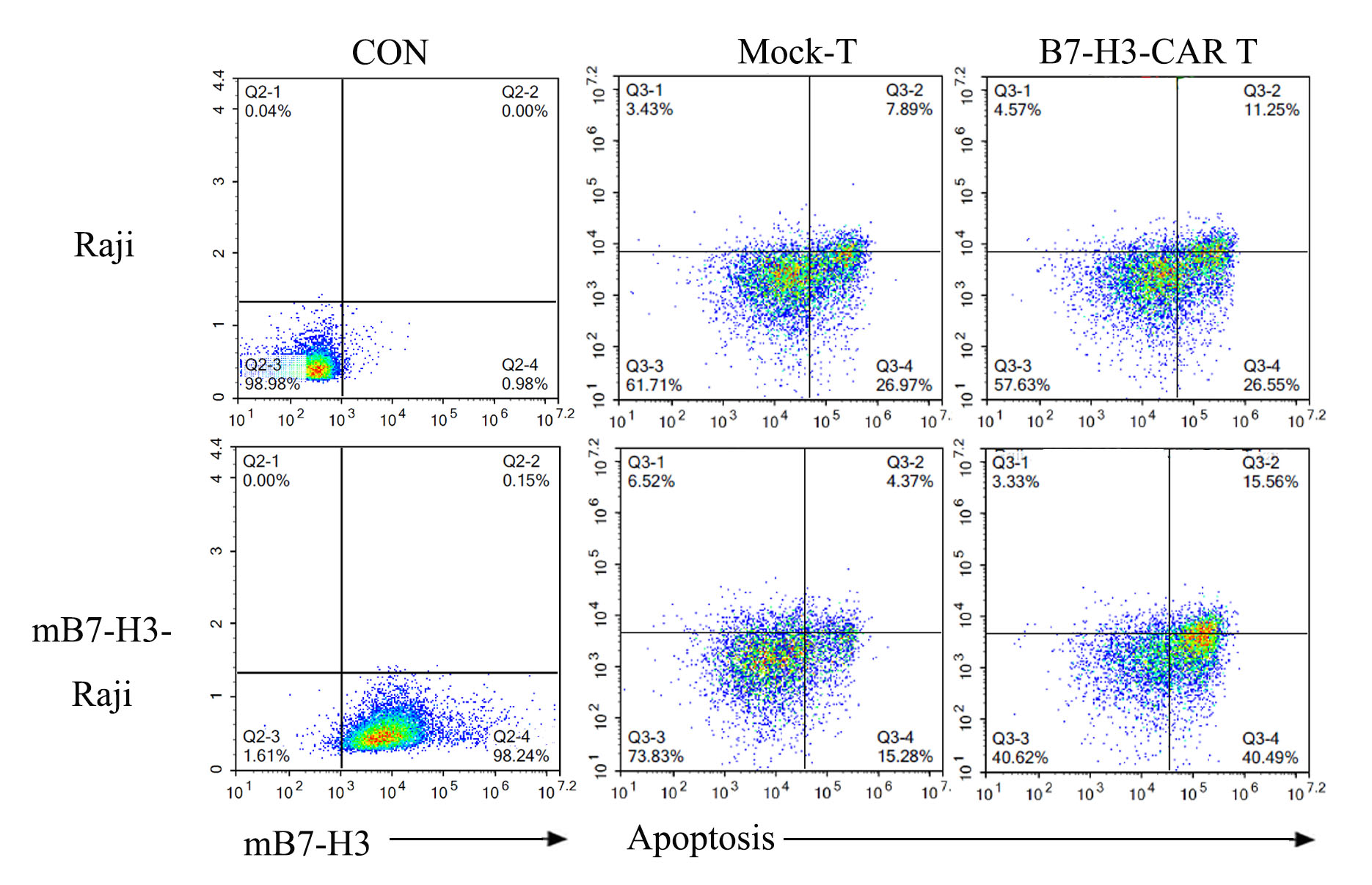

Supplement: Supplementary file 3 — Additional file 3. Apoptosis ratio of target cell detection following a 24h incubation under the effect-to-target ratio of 2:1. [file 40001_2023_1049_MOESM3_ESM.jpg]
